# Characterization of Shrimp Oil from *Pandalus borealis* by High Performance Liquid Chromatography and High Resolution Mass Spectrometry

**DOI:** 10.3390/md13063849

**Published:** 2015-06-18

**Authors:** Guangling Jiao, Joseph P. M. Hui, Ian W. Burton, Marie-Hélène Thibault, Claude Pelletier, Josée Boudreau, Nadia Tchoukanova, Balaji Subramanian, Yahia Djaoued, Stephen Ewart, Jacques Gagnon, Kathryn Vanya Ewart, Junzeng Zhang

**Affiliations:** 1 Coastal Zones Research Institute Inc., 232B, avenue de l’Église, Shippagan, NB E8S 1J2, Canada; E-Mails: Guangling.Jiao@nrc-cnrc.gc.ca (G.J.); marie-helene.thibault@umoncton.ca (M.-H.T.); claude.pelletier@umoncton.ca (C.P.); josee.boudreau@umoncton.ca (J.B.), jacques.gagnon@umoncton.ca (J.G.); 2 Natural Health Products Program, Aquatic and Crop Resource Development, National Research Council Canada, 1411 Oxford Street, Halifax, NS B3H 3Z1, Canada; E-Mails: Joseph.Hui@nrc-cnrc.gc.ca (J.P.M.H.); Ian.Burton@nrc-cnrc.gc.ca (I.W.B.); vewart@dal.ca (K.V.E.); 3 Université de Moncton, Campus de Shippagan, 218, boulevard J.-D.-Gauthier, Shippagan, NB E8S 1P6, Canada; E-Mails: subramanian.balaji@umoncton.ca (B.S.); yahia.djaoued@umoncton.ca (Y.D.); 4 Novaceutics Consulting, 6501 Oak St., Halifax, NS B3L 1H5, Canada; E-Mail: stephen.ewart@novaceutics.ca

**Keywords:** *Pandalus borealis*, shrimp oil, fatty acid profile, triacylglycerols, astaxanthin fatty acids esters, LC-HRMS and MS/MS, ^13^C-NMR

## Abstract

Northern shrimp (*Pandalus borealis*) oil, which is rich in omega-3 fatty acids, was recovered from the cooking water of shrimp processing facilities. The oil contains significant amounts of omega-3 fatty acids in triglyceride form, along with substantial long-chain monounsaturated fatty acids (MUFAs). It also features natural isomeric forms of astaxanthin, a nutritional carotenoid, which gives the oil a brilliant red color. As part of our efforts in developing value added products from waste streams of the seafood processing industry, we present in this paper a comprehensive characterization of the triacylglycerols (TAGs) and astaxanthin esters that predominate in the shrimp oil by using HPLC-HRMS and MS/MS, as well as ^13^C-NMR. This approach, in combination with FAME analysis, offers direct characterization of fatty acid molecules in their intact forms, including the distribution of regioisomers in TAGs. The information is important for the standardization and quality control, as well as for differentiation of composition features of shrimp oil, which could be sold as an ingredient in health supplements and functional foods.

## 1. Introduction

Northern shrimp (*Pandalus borealis*), the most abundant cold-water shrimp in the North Atlantic and Pacific areas, have been widely fished since the early 1900s. The Food and Agriculture Organization of the United Nations (FAO) situates their geographical distribution in the North Atlantic as ranging from Spitsbergen and Greenland south to the North Sea and to Massachusetts (U.S.A.), and in the North Pacific from the Bering Sea to southeast Siberia, Japan and Oregon (U.S.A.) [1]. The total global catch reported for this species since 2010 is between 315,511 to 446,909 t.

Heu *et al.* reported the components and nutritional quality of processing byproducts of Northern shrimps [2]. Crude protein content was in the range of 9.3%–11.6% and total fat content was about 0.7%. Rødde *et al.* compared seasonal variation of chemical composition of Northern shrimp shells [3]. The protein content was found to vary between 33% and 40% of the dry weight, while the chitin content varied between 17% and 20%. The shrimp shells had low lipid content, varying from 0.3% to 0.5% of the dry weight. This material is a rich source of nutrients such as chitin polymers [4,5], carotenoid pigments (mainly astaxanthin) [6,7] and polyunsaturated fatty acids (PUFAs) [8] that are valuable ingredients in the aquaculture industry.

Northern shrimp are usually peeled, cooked and frozen and sold in supermarkets, typically as appetizers. The processing waste consists of shells, heads and tails, representing approximately 50%–60% of the weight of the catch. The shrimp oil obtained as a byproduct from Northern shrimp processing facilities is rich in long-chained omega-3 fatty acids and has a bright red color attributed to the presence of astaxanthin.

Fatty acids (FAs) in marine oils are generally present in two forms, triacylglycerols and phospholipids (PLs). FAs found in krill oil are mainly as PL structures, whereas in fish oil they are exclusively as TAGs [9,10,11]. However, lipid profile of Northern shrimps has not been fully elucidated yet, except for two reports of *P. borealis* shrimp larvae [12,13]. Analysis revealed that PLs were the most abundant lipid class (~80% of total lipid weight) followed by TAGs (5%–15%).

Astaxanthin has been established as the most prevalent carotenoid in shrimp [14,15], representing about 65%–98% of the total carotenoids. It has potent biological activities against various diseases including cancer, hypertension, diabetes, cardiovascular, gastrointestinal, liver, neurodegenerative, and skin diseases [16,17]. Its antioxidant properties and uses have been investigated widely [18,19]. Approved by the United States Food and Drug Administration and the European Commission, astaxanthin has been used in nutrient supplementation and as a color additive in animal feed [16]. The increasing demand for its use in the fish farming industry and for human nutritional applications has led to commercial production of astaxanthin. Currently, commercial astaxanthin is mainly obtained from yeast and microalgae production and chemical synthesis. Shrimp processing byproducts are also an important source of astaxanthin [20,21]. Astaxanthin and its naturally formed esters are, however, usually present in low concentrations, and are often found in complex biological matrices [16], making its characterization challenging.

In the present work, we investigated the lipid classes, total profile of FAs and intact structural information of TAGs and astaxanthin esters of Northern shrimp oil derived from processing byproducts. In order to obtain a complete qualitative pattern of TAG molecules and astaxanthin esters, LC-HRMS and LC-MS/MS were used to separate and detect them without need for fractionation or derivatization of shrimp oil. Furthermore, tandem mass spectrometric data were obtained to confirm the fatty acids composition in individual TAGs and astaxanthin esters. On the other hand, the positions of fatty acids on TAG molecules were established by ^13^C-NMR. To our knowledge, this is the first comprehensive characterization of both TAGs and astaxanthin esters in Northern shrimp oil obtained from seafood processing, with new insight in regioisomeric distribution of fatty acids on TAG structures for this unique marine oil ingredient.

## 2. Results

### 2.1. Lipid Classification of Shrimp Oil by HPLC-CAD

**Figure 1:**
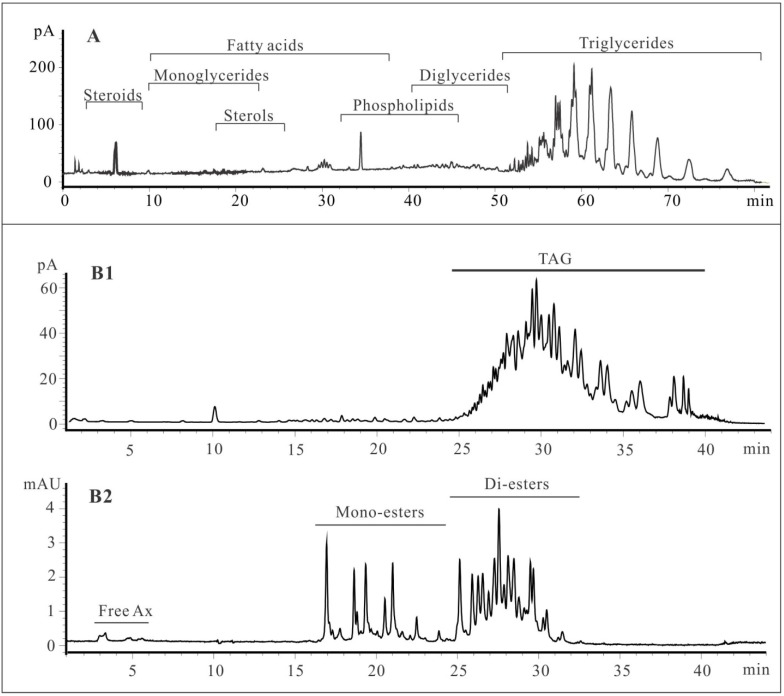
Characterization of shrimp oil on HPLC. **A**, Lipid classification of shrimp oil using gradient 1 detected by CAD detector. **B1**, Characterization of shrimp oil using gradient 2 by CAD detector; **B2**, Characterization of astaxanthin esters in shrimp oil using gradient 2 by DAD detector.

As shown in Figure 1A, the HPLC-CAD chromatogram revealed the presence of different lipid classes in shrimp oil. Polar lipids were eluted before 50 min, and TAG was eluted with a time window of 50–80 min. Although some polar lipids were visible, it is clear that TAGs represent the major composition of the shrimp oil.

Figure 1 B1 and B2 show typical chromatograms of shrimp oil on HPLC using gradient 2 detected by CAD detector and DAD (476 nm) detector, respectively. As shown in Figure 1 B1, a high abundance of triacylglycerol-type lipids was eluted at the end of the chromatogram, resulting in the pigments being only present as minor components in shrimp oil, approximately 1%, which suggested that a cleanup step prior to astaxanthin analysis was necessary. After the solid phase extraction (SPE) cleanup step, two fractions containing triacylglycerol and pigments, respectively, were obtained. The HPLC-CAD/DAD analysis (Figure S1) suggested that the chromatographic procedure had successfully removed all triacylglycerols from shrimp oil and provided astaxanthin-rich fraction for further analysis. Figure 1 B2 shows that the isomers of astaxanthin were eluted within the first six minutes. Due to the small amount of free astaxanthin in shrimp oil, it was not possible to elucidate the *trans*-/*cis*-forms of isomers based on the UV spectra. The second domain from 15 to 25 min was a group of singly esterified astaxanthin. Astaxanthin di-esters eluted between 25 to 35 min. The HPLC chromatogram (DAD 476 nm) showing astaxanthin molecules is divided into three groups that consisted of free astaxanthin (1.4%), astaxanthin monoesters (26.2%) and astaxanthin diesters (72.4%).

### 2.2. Fatty Acid Methylated Esters (FAMEs) Analysis by GC

The total fatty acids profile of TAG rich shrimp oil is shown in Table 1. Mean values of four replicates were presented. Some FAs such as 13:0, 20:0, 17:1*n*7, 16:4*n*3 and others are not detectable in all four experiments; thus, they may be present in trace amounts or be misidentified as a result of low intensity compared to signal noise. The results showed that fatty acids 18:1*n*9 (12.9%), 16:1*n*7 (12.5%), 16:0 (12.5%), 20:5*n*3 (eicosapentaenoic acid, EPA, 10.4%), 22:6*n*3 (docosahexaenoic acid, DHA, 9.5%) and 22:1*n*9 (7.7%) were the most abundant fatty acids in shrimp oil. The amount of PUFA (29.2%) and saturated fatty acids (19.7%) are lower than that of monounsaturated fatty acids (MUFA) (49.9%). In addition, the PUFAs were high in ω-3 PUFAs (25.2%) and low in ω-6 PUFAs (1.7%). The findings from *P. borealis* shrimp oil are consistent with that of whole *P. borealis* shrimp, as shown in Table 1 [22].

**Table 1:** Fatty Acid Methylated Esters (FAME) profiles of shrimp oil by GC analysis.

FAME	*P. borealis* Shrimp Oil		*P. borealis* Whole Shrimp [22]	Krill Oil [23]	
	Intensity	RSD% (*n* = 4)		Original Oil	TAG Rich Fraction
**12:0**	0.2%	30.8		0.1% ± 0.0%	0.2% ± 0.0%
**13:0**	Trace				
**14:0**	3.6%	19.1	2.3%	1.1% ± 0.0%	17.5% ± 0.2%
**15:0**	0.3%	15.9		0.4% ± 0.0%	0.4% ± 0.0%
**16:0**	12.5%	2.1	14.9%	24.0% ± 0.1%	18.9% ± 0.2%
**17:0**	0.2%	32.9		0.2% ± 0.0%	0.2% ± 0.0%
**18:0**	2.9%	17.5	2.9%	1.0% ± 0.0%	0.8% ± 0.0%
**20:0**	Trace			0.1% ± 0.0%	0.1% ± 0.0%
**22:0**	Trace				
**14:1*n*5**	0.2%	17.3			
**16:1*n*5**	0.4%	22.5			
**16:1*n*7**	12.5%	13.2	14.5%	5.5% ± 0.0%	9.2% ± 0.2%
**17:1*n*7**	Trace				
**18:1*n*5**	0.7%	15.0			
**18:1*n*7**	4.3%	10.8	7.7%	6.6% ± 0.1%	6.3% ± 0.1%
**18:1*n*9**	12.9%	7.3	12.3%	10.0% ± 0.0%	10.9% ± 0.1%
**20:1*n*7**	1.7%	49.3		0.2% ± 0.0%	0.2% ± 0.0%
**20:1*n*9**	6.3%	36.7	2.7%	0.6% ± 0.0%	0.5% ± 0.0%
**20:1*n*11**	0.8%	-			
**22:1*n*7**	2.4%	56.2			
**22:1*n*9**	7.7%	39.0	1.3%	0.6% ± 0.0%	0.3% ± 0.0%
**24:1*n*9**	Trace			0.1% ± 0.0%	
**16:2*n*4**	0.5%	53.3		0.5% ± 0.0%	1.1% ± 0.0%
**16:2*n*6**	0.3%	81.5			
**16:3*n*3**	0.2%	16.7		0.1% ± 0.0%	2.5% ± 0.0%
**16:3*n*4**	0.4%	37.1			
**16:4*n*1**	0.3%	4.9			
**16:4*n*3**	Trace			0.8% ± 0.0%	
**18:2*n*4**	0.4%	28.3			
**18:2*n*6**	0.8%	8.4	0.6%	1.7% ± 0.0%	1.7% ± 0.0%
**18:3*n*3**	0.6%	44.0	Trace	1.3% ± 0.0%	1.4% ± 0.0%
**18:3*n*4**	0.7%	-			
**18:3*n*6**	Trace			0.2% ± 0.0%	0.3% ± 0.0%
**18:4*n*1**	Trace				
**18:4*n*3**	1.1%	8.2	0.5%	3.8% ± 0.1%	6.8% ± 0.1%
**20:2*n*6**	0.3%	38.8			
**20:2*n*9**	Trace				
**20:3*n*3**	0.2%	73.5			
**20:3*n*6**	Trace			0.0% ± 0.0%	
**20:4*n*3**	2.4%	-	Trace	0.4% ± 0.0%	0.3% ± 0.0%
**20:4*n*6**	0.4%	34.2		0.4% ± 0.0%	0.3% ± 0.0%
**20:5*n*3**	10.4%	11.3	12.5%	18.5% ± 0.2%	9.7% ± 0.0%
**21:5*n*3**	0.3%	23.0		0.6% ± 0.0%	0.3% ± 0.0%
**22:2**	Trace				
**22:4*n*6**	Trace				
**22:5*n*3**	0.5%	7.2	1.0%	0.4% ± 0.0%	0.2% ± 0.0%
**22:5*n*6**	Trace			0.0% ± 0.0%	
**22:6*n*3**	9.5%	7.1	7.7%	9.0% ± 0.0%	3.8% ± 0.0%
**Total**	98.8%	0.00			
**Saturated (SFAs)**	19.7%	3.4	21.4%	38.1% ± 0.0%	43.4% ± 0.0%
**Monounsaturated (MUFAs)**	49.9%	8.8	46.0%	24.0% ± 0.2%	27.9% ± 0.1%
**Polyunsaturated (PUFAs)**	29.2%	16.1	25.3%	37.9% ± 0.2%	28.8% ± 0.1%
**Omega-3**	25.2%	18.9	21.7%	35.0% ± 0.2%	25.2% ± 0.1%
**Omega-6**	1.7%	17.7	0.6%	2.3% ± 0.0%	2.3% ± 0.0%

### 2.3. Characterization of TAGs in Shrimp Oil

#### 2.3.1. Identification of TAGs Species by LC-HRMS

**Figure 2:**
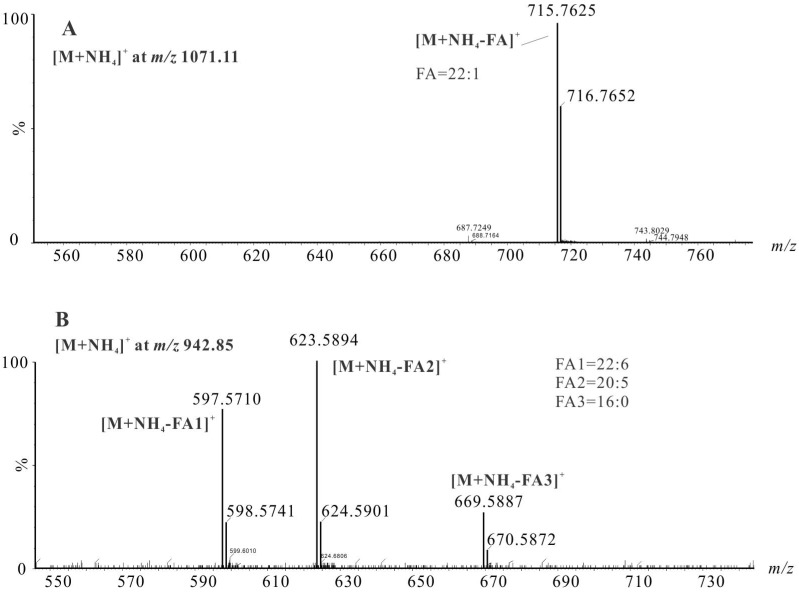
MS/MS spectra of [M + NH_4_]^+^ adducts of TAGs in shrimp oil at *m/z* 1071.11 (**A**) and 942.85 (**B**).

Data-dependent acquisition was then employed to characterize the fatty acids profiles on TAG backbone. Typical data used for TAG identification are shown in Figure 2. According to MacDougall *et al.*, diacylglycerol (DAG) fragment ions yielded by an intact TAG indicated the neutral loss of fatty acids from the glycerol backbone [24]. A TAG molecule with the same fatty acid on its backbone exhibited a simple MS/MS spectrum with only one single DAG fragment ion. For instance, the ammoniated ion [M + NH_4_]^+^ at *m/z* 1071.11 gave one DAG ion at *m/z* 715.76 (Figure 2A), resulting neutral loss of fatty acid 22:1 from TAG(22:1/22:1/22:1). Fragmentation of *m/z* 942.85 (Figure 2B) resulted in three DAG fragment ions at *m/z* 597.57, 623.59 and 669.59 corresponding to the neutral loss of a fatty acid 22:6, 20:5 and 16:0, indicating that TAG(58:11) was TAG(22:6/20:5/16:0).

**Figure 3:**
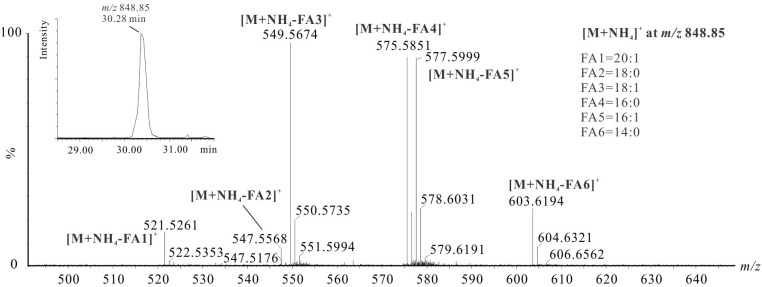
LC-MS/MS spectrum of the ammoniated adducts at *m/z* 848.85 from shrimp oil TAG(50:2) at 30.29 min. The MS/MS interpretation of this precursor ion suggested contributions of four TAG species, namely TAG(16:0/16:1/18:1) (major), TAG(14:0/16:1/20:1), TAG(14:0/18:1/18:1) and TAG(16:1/16:1/18:0).

In some occurrences, LC-MS/MS analysis of shrimp oil also revealed the presence of structural isomers due to the potentially co-eluting TAGs. For instance, although the [M + NH_4_]^+^ ion at *m/z* 848.85 exhibits one chromatographic peak at 30.28 min (Figure 3), the detection of six DAG fragment ions at *m/z* 521.53, 547.56, 549.57, 575.59, 577.60 and 603.62 resulted from the dissociation of fatty acids 20:1, 18:0. 18:1, 16:0, 16:1 and 14:0 of TAG(50:2) backbone, which suggests four TAG species namely TAG(16:0/16:1/18:1) (major), TAG(14:0/16:1/20:1), TAG(14:0/18:1/18:1) and TAG(16:1/16:1/18:0). Similarly, the identification of other co-eluting ammoniated TAG isomers from single chromatographic peaks is presented. Collectively, an overview of the interpretation of intact TAG molecules of shrimp oil determined by LC-MS/MS is provided in Table 2. As a result, a total of 118 TAGs species was characterized from shrimp oil. Their relative abundance was obtained based on their MS peak area. As listed in Table 2, some of the most abundant TAGs in shrimp oil include TAG(16:0/16:1/16:1), TAG(16:0/20:1/20:5), TAG(16:0/18:1/20:5), TAG(16:1/18:1/18:1), TAG(16:1/16:1/20:1), TAG(18:1/18:1/22:6), TAG(16:0/16:0/20:5), TAG(16:1/22:1/22:6), TAG(18:1/20:1/22:6). It showed that at the molecular level, the omega-3 fatty acids such as DHA and EPA were on TAG backbones with higher levels of MUFAs (16:1, 18:1, 20:1 and 22:1) than SFAs (14:0 and 16:0). Also, when DHA and EPA were found on the same TAG structure, the other fatty acids were likely to be 16:0, 16:1 or 18:1. To our knowledge, this is the first report of a full elucidation of TAG species in shrimps.

**Table 2:** Characterization of triacylglycerols (TAGs) in shrimp oil by LC-HRMS and MS/MS.

No.	Molecular Formula	RT (min)	Calculated *m/z* [M + NH_4_]^+^	Observed [M + NH_4_]^+^	C:DB	Intensity (%)	TAG Identity	
							Major Composition	Other Composition
1	C49H90O6	27.79	792.7076	792.7081	TAG(46:2)	1.4	TAG(14:0/16:1/16:1)	TAG(12:0/16:1/18:1)
2	C49H92O6	28.70	794.7232	794.7256	TAG(46:1)	3.2	TAG(14:0/16:0/16:1)	TAG(14:0/14:0/18:1), TAG(12:0/16:0/18:1)
3	C51H92O6	28.31	818.7232	818.7220	TAG(48:3)	2.5	TAG(16:1/16:1/16:1)	TAG(14:1/16:1/18:1)
4	C51H94O6	29.13	820.7389	820.7413	TAG(48:2)	6.4	TAG(16:0/16:1/16:1)	TAG(14:0/16:1/18:1)
5	C51H96O6	29.90	822.7545	822.7618	TAG(48:1)	1.3	TAG(16:0/16:0/16:1)	TAG(14:0/16:0/18:1)
6	C53H90O6	27.40	840.7053	840.7105	TAG(50:6)	1.4	TAG(14:0/16:1/20:5)	
7	C53H96O6	29.54	846.7545	846.7565	TAG(50:3)	2.9	TAG(16:1/16:1/18:1)	TAG(14:1/18:1/18:1), TAG(14:1/16:1/20:1)
8	C53H98O6	30.29	848.7702	848.7761	TAG(50:2)	1.6	TAG(16:0/16:1/18:1)	TAG(14:0/16:1/20:1), TAG(14:0/18:1/18:1), TAG(16:1/16:1/18:0)
9	C53H100O6	31.11	850.7858	850.7929	TAG(50:1)	2.3	TAG(16:0/16:0/18:1)	TAG(14:0/16:0/20:1)
10	C55H92O6	27.89	866.7232	866.7246	TAG(52:7)	2.4	TAG(16:1/16:1/20:5)	
11	C55H94O6	28.70	868.7389	868.7418	TAG(52:6)	2.7	TAG(16:0/16:1/20:5)	TAG(14:0/18:1/20:5)
12	C55H96O6	29.62	870.7545	870.7586	TAG(52:5)	3.0	TAG(16:0/16:0/20:5)	TAG(14:0/18:0/20:5)
13	C55H100O6	30.75	874.7858	874.7930	TAG(52:3)	3.4	TAG(16:1/18:1/18:1), TAG(16:1/16:1/20:1)	TAG(14:1/18:1/20:1), TAG(14:1/16:1/22:1)
14	C55H102O6	31.44	876.8015	876.8102	TAG(52:2)	1.4	TAG(16:0/16:1/20:1), TAG(16:0/18:1/18:1)	TAG(14:0/18:1/20:1), TAG(14:0/16:1/22:1), TAG(16:1/18:0/18:1)
15	C55H104O6	32.24	878.8171	878.8104	TAG(52:1)	1.3	TAG(16:0/18:0/18:1)	TAG(14:0/16:0/22:1), TAG(14:0/18:0/20:1), TAG(16:0/16:0/20:1)
16	C57H90O6	26.97	888.7076	888.7070	TAG(54:10)	0.6	TAG(14:0/20:5/20:5)	
17	C57H96O6	29.22	894.7545	894.7576	TAG(54:7)	2.8	TAG(16:1/18:1/20:5)	TAG(16:0/16:1/22:6), TAG(14:0/18:1/22:6)
18	C57H98O6	30.00	896.7702	896.7792	TAG(54:6)	4.5	TAG(16:0/18:1/20:5)	TAG(14:0/20:1/20:5)
19	C57H104O6	31.88	902.8171	902.8209	TAG(54:3)	2.6	TAG(16:1/18:1/20:1), TAG(18:1/18:1/18:1)	TAG(16:1/16:1/22:1), TAG(14:1/20:1/20:1), TAG(14:1/18:1/22:1)
20	C57H106O6	32.57	904.8328	904.8364	TAG(54:2)	2.3	TAG(16:0/18:1/20:1)	TAG(14:0/20:1/20:1), TAG(16:0/16:1/22:1), TAG(14:0/18:1/22:1), TAG(16:1/18:0/20:1), TAG(18:0/18:1/18:1)
21	C59H92O6	27.41	914.7232	914.7269	TAG(56:11)	1.6	TAG(16:1/20:5/20:5)	TAG(16:1/18:4/22:6)
22	C59H98O6	29.75	920.7702	920.7770	TAG(56:8)	2.2	TAG(16:1/18:1/22:6)	
23	C59H100O6	30.37	922.7858	922.7889	TAG(56:7)	2.3	TAG(18:1/18:1/20:5)	TAG(16:0/18:1/22:6), TAG(16:1/20:1/20:5)
24	C59H102O6	31.22	924.8015	924.8085	TAG(56:6)	3.5	TAG(16:0/20:1/20:5)	TAG(18:0/18:1/20:5), TAG(18:1/18:4/20:1)
25	C59H108O6	32.93	930.8484	930.8499	TAG(56:3)	2.5	TAG(16:1/18:1/22:1), TAG(16:1/20:1/20:1), TAG(18:1/18:1/20:1)	TAG(14:1/20:1/22:1), TAG(14:1/18:1/24:1)
26	C59H110O6	33.59	932.8641	932.8682	TAG(56:2)	2.6	TAG(16:0/18:1/22:1), TAG(16:0/20:1/20:1)	TAG(14:0/20:1/22:1), TAG(16:1/18:0/22:1), TAG(18:0/18:1/20:1)
27	C61H94O6	28.06	940.7389	940.7376	TAG(58:12)	1.0	TAG(16:1/20:5/22:6)	
28	C61H96O6	28.96	942.7545	942.7576	TAG(58:11)	1.7	TAG(16:0/20:5/22:6)	
29	C61H102O6	30.99	948.8015	948.8088	TAG(58:8)	3.3	TAG(18:1/18:1/22:6)	TAG(16:1/20:1/22:6)
30	C61H104O6	31.53	950.8171	950.8204	TAG(58:7)	1.9	TAG(16:1/20:5/22:1)	TAG(18:1/20:1/20:5)
31	C61H106O6	32.30	952.8328	952.8352	TAG(58:6)	2.4	TAG(16:0/20:5/22:1)	TAG(18:0/20:1/20:5)
32	C61H114O6	34.57	960.8954	960.8954	TAG(58:2)	2.6	TAG(16:0/20:1/22:1)	TAG(14:0/20:1/24:1), TAG(14:0/22:1/22:1), TAG(16:0/18:1/24:1), TAG(18:0/18:1/22:1), TAG(18:0/20:1/20:1)
33	C63H98O6	29.30	968.7702	968.7745	TAG(60:12)	0.9	TAG(18:1/20:5/22:6)	
34	C63H106O6	32.11	976.8328	976.8328	TAG(60:8)	2.9	TAG(16:1/22:1/22:6),TAG(18:1/20:1/22:6)	
35	C63H108O6	32.66	978.8484	978.8484	TAG(60:7)	2.8	TAG(18:1/20:5/22:1)	TAG(20:1/20:1/20:5)
36	C63H110O6	33.40	980.8641	980.8679	TAG(60:6)	0.7	TAG(18:0/20:5/22:1)	
37	C63H116O6	34.85	986.9110	986.9110	TAG(60:3)	1.9	TAG(16:1/22:1/22:1), TAG(18:1/20:1/22:1)	TAG(16:1/20:1/24:1), TAG(18:1/18:1/24:1),
38	C63H118O6	35.47	988.9267	988.9183	TAG(60:2)	1.4	TAG(16:0/22:1/22:1)	TAG(16:0/20:1/24:1), TAG(14:0/22:1/24:1), TAG(18:0/20:1/22:1)
39	C65H110O6	33.13	1004.8641	1004.8661	TAG(62:8)	2.4	TAG(18:1/22:1/22:6), TAG(20:1/20:1/22:6)	
40	C65H112O6	33.63	1006.8797	1006.8805	TAG(62:7)	1.0	TAG(20:1/20:5/22:1)	TAG(18:1/20:5/24:1)
41	C65H120O6	35.74	1014.9423	1014.9392	TAG(62:3)	2.3	TAG(18:1/22:1/22:1), TAG(20:1/20:1/22:1)	TAG(16:1/22:1/24:1), TAG(18:1/20:1/24:1)
42	C67H114O6	34.06	1032.8954	1032.8907	TAG(64:8)	1.3	TAG(20:1/22:1/22:6)	
43	C67H124O6	36.62	1042.9736	1042.9720	TAG(64:3)	2.3	TAG(20:1/20:1/24:1)	TAG(18:1/22:1/24:1), TAG(20:1/22:1/22:1)
44	C69H118O6	35.01	1060.9267	1060.9216	TAG(66:8)	1.3	TAG(22:1/22:1/22:6)	
45	C69H128O6	37.41	1071.0049	1071.0076	TAG(66:3)	1.5	TAG(22:1/22:1/22:1)	TAG(20:1/22:1/24:1)

**Figure 4:**
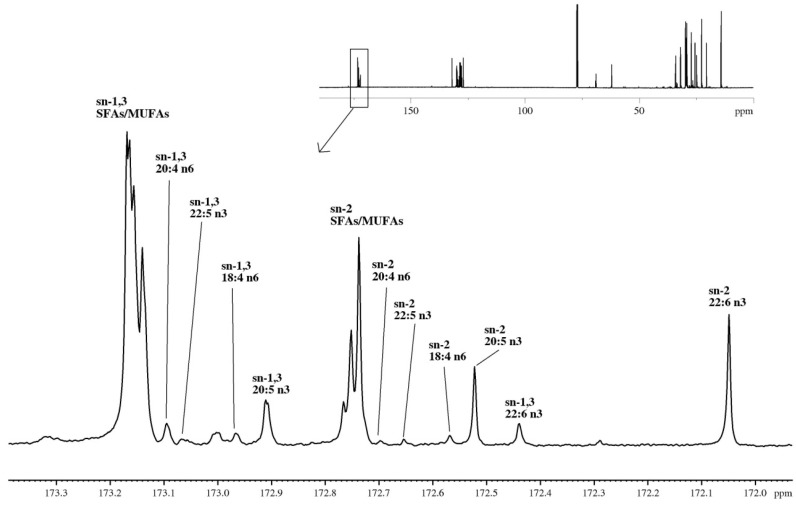
^13^C-NMR spectra of shrimp oil, with the carbonyl region (172.0–173.4 ppm) enlarged. The assignment of *sn*-1,3 and *sn*-2 regioisomeric peaks to individual fatty acids is annotated.

**Table 3:** Regioisomeric distribution (molar %) of major fatty acids on TAG molecules in shrimp oil by ^13^C-NMR.

Source	Molar (%)	*sn*-1,3	*sn*-2	Total
*P. borealis* Shrimp oil	SFAs + MUFAs	56.8	19.2	75.9
	20:5*n*3 (EPA)	4.1	4.5	9.0
	22:6*n*3 (DHA)	1.2	7.1	8.3
	Omega-3			17.8
	Omega-6			2.8
Cod liver oil [27]	EPA	2.1	6.4	8.5
	DHA	0.4	9.2	9.6
Tuna oil [28]	EPA		6.7	
	DHA		11.5	
Algae oil [28]	DHA		35.2	
Anchovy/sardine fish oil [25]	EPA	80.9	19.1	
	DHA	38.3	61.7	

#### 2.3.2. Regioisomeric Distributions of Fatty Acids on TAGs Determined by ^13^C-NMR

The proportion of major fatty acids attached to the *sn*-1,3 and *sn*-2 positions of TAGs in shrimp oil was determined using a ^13^C-NMR method as described in previous reports [25,26]. As shown in Figure 4, the carbonyl region of the broadband decoupled ^13^C-NMR spectrum is displayed. Chemical shifts starting from the highest frequency peak at 173.2 ppm were assigned to SFA and MUFA chains attached to *sn*-1,3 positions. The progressive decreasing chemical shifts of PUFAs on *sn*-1,3 chains were then assigned. As published, carbonyl carbons of *sn*-1,3 and *sn*-2 acyl chains were separated by a systematic shift of about 0.4 ppm forming clusters of *sn*-1,3 and *sn*-2 peaks [25,26]. Thus, the chemical shifts starting at 172.8 ppm were then assigned as SFAs and MUFAs attached to *sn*-2 positions. The regioisomeric distributions (molar %) of major fatty acids on TAG molecules in shrimp oil are listed in Table 3. The SFAs and MUFAs showed slightly preferable residence in the *sn*-1,3 position of 56.8%, whereas DHA and EPA were preferentially attached to the *sn*-2 position. The FAs 20:4*n*6, 22:5*n*3 and 18:4*n*6 attached on different positions at TAG molecules were also identified by chemical shift on ^13^C-NMR in Figure 4, but their distributions (molar %) were not reported here due to their very low intensities.

### 2.4. Characterization of Astaxanthin and Its Esters in Shrimp Oil

#### 2.4.1. Identification of Astaxanthin and Its Esters by LC-HRMS

Initial screening for astaxanthin esters was carried out by LC-HRMS. This eliminates the need for sample derivatization or fractionation. Free astaxanthin molecules were shown as protonated species [M + H] ^+^ at *m/z* 597.3934. For examples of astaxanthin esters, a monoester (Ax-C22:6) at *m/z* 907.6215 and a diester (Ax-C40:7) at *m/z* 1171.8650 were depicted. The Exactive™ MS spectra cannot give the specific fatty acid information on astaxanthin, thus LC-MS/MS data are needed to distinguish them. As the total ion LC-MS chromatogram of shrimp oil suggested the column was overloaded with triacylglycerol, therefore the astaxanthin-rich fraction after clean-up step has been applied for LC-MS/MS analysis.

**Figure 5:**
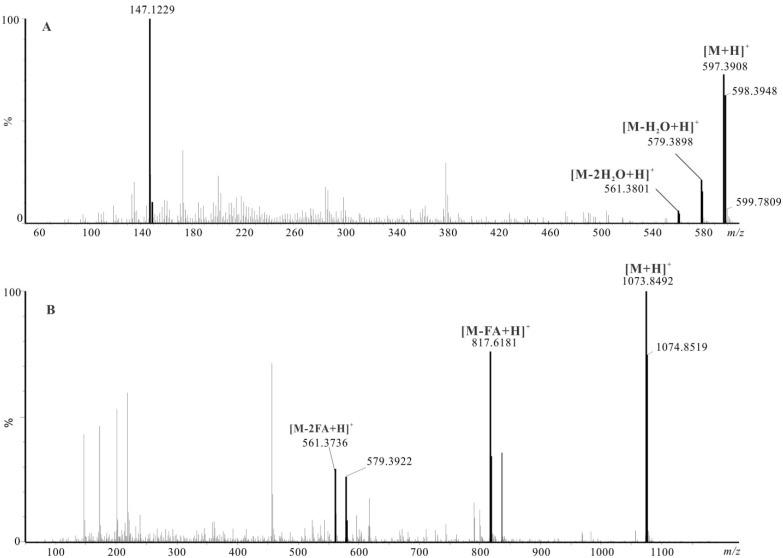
LC-MS/MS of astaxanthin standard (**A**) and astaxanthin 16:0/16:0 di-ester (**B**). FA represents fatty acid.

Tandem mass spectrometry provided further structural confirmation of astaxanthin mono-esters and di-esters. For example, the MS/MS spectrum of astaxanthin and its 16:0/16:0 di-ester standards are presented in Figure 5A,B, respectively. Fragmentation of astaxanthin is characterized by the loss of two water molecules, resulting in *m/z* 579.3898 and *m/z* 561.3801. On the other hand, fragmentation of the 16:0/16:0 di-ester is characterized by the neutral loss of its palmitic acid, resulting in the prominent fragments at *m/z* 817.6181 and *m/z* 561.3736. Frassanito *et al.* reported using the fragment at *m/z* 579.4 to represent a “true” signature of the astaxanthin backbone, while Rivera *et al.* also used the same water loss fragment to carry out quantification [29,30].

**Figure 6:**
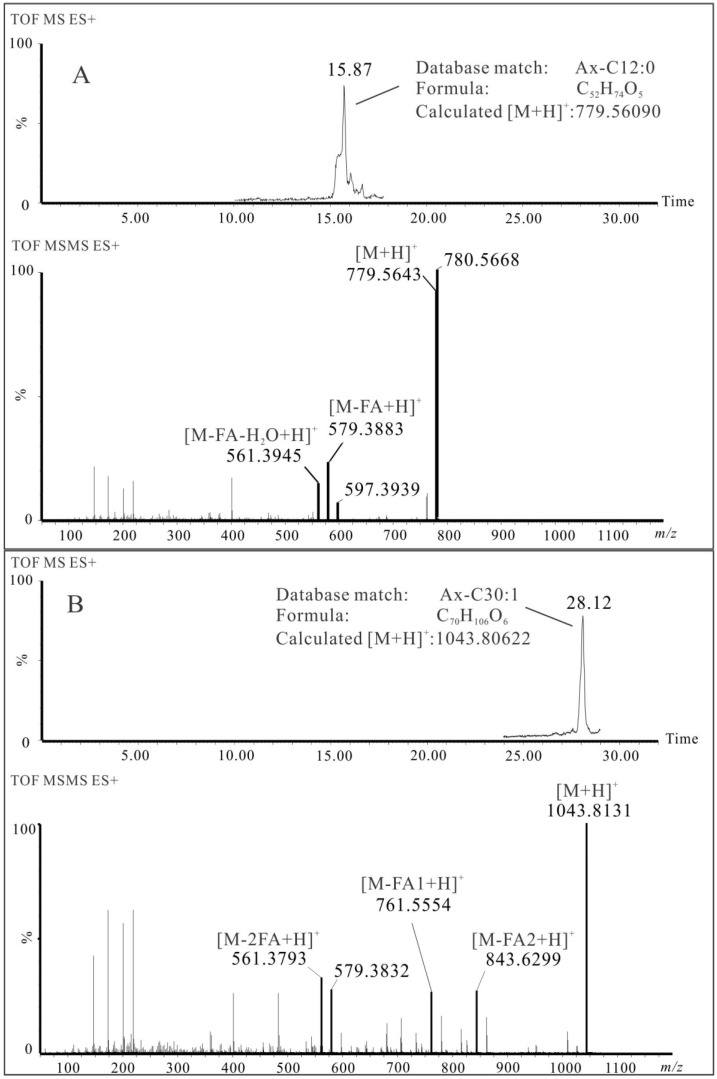
Examples of LC-MS/MS of an astaxanthin 12:0 mono-ester (**A**) and an astaxanthin 12:0/18:1 di-ester (**B**) from shrimp oil.

Examples of MS/MS spectra of Ax-C12:0 and Ax-C12:0/18:1 detected in shrimp oil are shown in Figure 6. The neutral loss of the fatty acid C12:0 from its astaxanthin mono-ester at *m/z* 779.5643 resulted in the fragment at *m/z* 579.3883 (Figure 6A). In the case of the astaxanthin di-ester at *m/z* 1043.8131, two fragments at *m/z* 843.6299 and 761.5554 corresponded to the neutral loss of fatty acid C12:0 and C18:1, respectively, while the fragment at *m/z* 561.3793 resulted from the loss of both fatty acids (Figure 6B). There are instances where co-elution of di-esters with different compositions of fatty acids was observed. An example is Ax-C28:0, whereby tandem MS resulted in fragments *m/z* 761.6426, 789.6770 and 817.6915, which indicated the presence of Ax-C12:0/C16:0 and Ax-C14:0/C14:0. In summary, a total of nine astaxanthin mono-esters and 26 di-esters were confirmed. The fragments related to neutral loss of fatty acids from the astaxanthin esters are summarized in Table 4. The diagnostic fragments at *m/z* 579.4 and *m/z* 561.4 were detected in all of them.

As displayed in Table 4, astaxanthin di-esters are observed as the main derivatives. This result was consistent with the composition obtained by HPLC-DAD analysis, while providing detailed structural information. Furthermore, lauric acid (C12:0) and oleic acid (C18:1) were found to be the prevalent fatty acids in astaxanthin esters in shrimp oil. PUFAs, particularly EPA and DHA esters such as Ax-C20:5 (5.1% of total astaxanthin compounds), Ax-C22:6 (2.3%), Ax-C12:0/20:5 (4.9%), Ax-C12:0/22:6 (2.6%), Ax-C20:5/20:5 (1.3%), Ax-C20:5/22:6 (1.1%), Ax-C16:1/20:5 (2.2%), Ax-C16:0/20:5 (2.3%), Ax-C18:1/20:5 (5.5%), Ax-C18:1/22:6 (2.0%) and Ax-C20:1/20:5 (1.3%) were also present. Compared with reported astaxanthin compounds in *P. borealis* shrimp and other species [31,32,33], Ax-C16:1/20:5, Ax-C20:1/20:5, Ax-C14:0/20:1, Ax-C16:1/20:1, Ax-C16:0/20:1, Ax-C20:1/20:1 and Ax-C18:1/22:1 were found to be unique in the shrimp oil we analyzed.

## 3. Discussion

### 3.1. Lipid Classes and FA Profiles of Shrimp Oil

An HPLC method using a universal CAD detector coupled with a HALO C8 column has been optimized [34]. This method can separate and identify different lipid classes including free fatty acids (lauric to stearic acid), fatty acid-esters and alcohols, phospholipids, acylated glycerols (mono-, di-, and tri-acylglycerols and milk fats), and paraffins (octadecane to octacosane). Large amounts of TAGs were unquestionably present in the shrimp oil characterized by HPLC-CAD. Trace amount of polar lipids also exists. In contrast with shrimp oil, krill oil from *Euphausia superba* contained predominantly phospholipids as shown in Table 5 [11,35]. The lipid variation may be caused by the species, state of processing (whole organism or processed), environment and life stage, as well as extraction method.

**Table 4:** Identification of astaxanthin fatty acids esters in shrimp oil by LC Q-TOF MS/MS spectra.

Compounds		Molecular Formula	RT (min)	Intensity (%)	Observed *m/z* on MS/MS					Identification by Other Reports			
					[M + H]^+^	[M-FA1 + H]^+^	[M-FA2 + H]^+^	Ax Backbone Related MS/MS		*P. borealis* [31]	*L. vannamei* [32]	Krill [33]	Microalgae [31]
**Free Ax**													
1	Astaxanthin	C40H52O4	2.10	0.5	597.3663			579.3843	561.4108				
2	Astaxanthin isomer1	C40H52O4	3.56	0.5	597.3266			579.3949	561.3801				
3	Astaxanthin isomer2	C40H52O4	3.85	0.4	597.3575			579.3616	561.3696				
**Ax-FA Monoesters**													
4	Ax-C12:0	C52H74O5	15.87	7.3	779.5643	579.3883			561.3945	✓		✓	
5	Ax-C20:5	C60H80O5	17.78	5.1	881.6111	579.3793			561.3813		✓		
6	Ax-C14:0	C54H78O5	17.80	1.3	807.6991	579.4027			561.4552	✓		✓	
7	Ax-C22:6	C62H82O5	18.56	2.3	907.7129	579.4470			561.4552		✓		
8	Ax-C16:1	C56H80O5	18.41	2.3	833.6066	579.3861			561.3824			✓	
9	Ax-C16:0	C56H82O5	19.63	2.4	835.7094	579.4558			561.4291	✓	✓	✓	✓
10	Ax-C18:1	C58H84O5	20.18	3.9	861.6601	579.3837			561.3689	✓	✓	✓	✓
11	Ax-C18:0	C58H86O5	21.39	0.9	863.6774	579.4111			561.3632		✓	✓	✓
12	Ax-C20:1	C60H88O5	21.82	2.0	889.7763	579.4647			561.4116	✓			
**Ax-FA Diesters**													
13	Ax-C12:0/20:5	C72H102O6	26.47	4.9	1063.8861	863.6884	761.6426	579.447	561.4377			✓	
14	Ax-C12:0/22:6	C74H104O6	26.99	2.6	1089.8059	889.5828	761.5556	579.384	561.3867			✓	
15	Ax-C20:5/20:5	C80H108O6	27.51	1.3	1165.8419	863.5995	863.5995	579.3549	561.3832		✓		
16	Ax-C12:0/16:0	C68H104O6	27.78	5.8	1017.8812	817.6915	761.6426	579.4558	561.4291	✓		✓	
17	Ax-C14:0/14:0					789.6770	789.6770					✓	
18	Ax-C20:5/22:6	C82H110O6	28.02	1.1	1191.8425	889.6176	863.5938	579.3936	561.3767		✓		
19	Ax-C16:1/20:5	C76H108O6	28.04	2.2	1117.8411	863.5801	815.6221	579.3740	561.3672				
20	Ax-C12:0/18:1	C70H106O6	28.12	12.6	1043.8131	843.6299	761.5554	579.3832	561.3793	✓		✓	
21	Ax-C14:0/16:1					815.6266	789.5727					✓	
22	Ax-C16:0/20:5	C76H110O6	28.76	2.3	1119.8601	863.6044	817.5956	579.3857	561.3710		✓		
23	Ax-C18:1/20:5	C78H112O6	29.13	5.5	1145.8514	863.5996	843.6376	579.3837	561.3719		✓	✓	
24	Ax-C16:0/22:6					889.5994	817.6177				✓		
25	Ax-C12:0/20:1	C72H110O6	29.23	7.3	1071.8407	871.6646	761.5882	579.3846	561.3730	✓		✓	
26	Ax-C14:0/18:1					843.6436	789.5870					✓	
27	Ax-C16:1/18:1	C74H112O6	29.56	4.4	1097.9844	843.7114	815.6818	579.4558	561.4291			✓	
28	Ax-C18:1/22:6	C80H114O6	29.78	2.0	1171.8672	889.6319	843.6218	579.3994	561.3832		✓	✓	
29	Ax-C20:1/20:5	C80H116O6	30.17	1.3	1173.8394	863.6236	871.6613	579.3936	561.3782				
30	Ax-C16:0/18:1	C74H114O6	30.26	6.3	1099.8682	843.6331	817.6174	579.3845	561.3745		✓	✓	
31	Ax-C14:0/20:1					871.6799	789.6058						
32	Ax-C18:1/18:1	C76H116O6	30.57	5.4	1125.9897	843.7221	843.7221	579.4470	561.4377	✓		✓	✓
33	Ax-C16:1/20:1					871.7469	815.7239						
34	Ax-C16:0/20:1	C76H118O6	31.25	2.0	1127.9919	871.7578	817.7126	579.4558	561.4203				
35	Ax-C18:0/18:1					843.7327	845.7125						✓
36	Ax-C18:1/20:1	C78H120O6	31.55	2.8	1153.9973	871.7469	843.7006	579.4558	561.4465	✓			
37	Ax-C20:1/20:1	C80H124O6	32.49	1.3	1181.9945	871.7469	871.7469	579.4558	561.4291				
38	Ax- C18:1/22:1					843.7114	899.8098						

**Table 5:** Lipid contents (percentage of total lipids) in different sources.

Sources	Phospholipids	Triacylglycerols	Other Polar Lipids
*P. borealis* shrimp oil		>90%	
*P. borealis* shrimp larvae [12,13]	~80%	5%–15%	
Krill oil [11]	20%–33%	1%–3%	64%–77%
Krill oil [35]	93.1%	1.7%	5.4%
Krill oil [35]	51.3%	33.4%	15.2%

Since TAG is the main component in shrimp oil, the fatty acid profiles obtained by GC analysis can reflect the native pattern of FA esterified with TAG molecules of shrimp oil, which is composed of 19.7% of SFAs, 49.9% of MUFAs and 29.2% of PUFAs. Fatty acids 18:1*n*9, 16:1*n*7, 16:0, 20:5*n*3, 22:6*n*3 and 22:1*n*9 were the most abundant fatty acids in shrimp oil. This result is in a similar trend with the report by Dahl *et al.*’s on the fatty acid composition in whole *P. borealis* shrimps containing 21.4% of SFAs, 46.0% of MUFAs and 25.3% of PUFAs [22]. In his work, 16:0 (14.9%), 16:1*n*7 (14.5%), 20:5*n*3 (12.5%), 18:1*n*9 (12.3%), 18:1*n*7 (7.7%) and 22:6*n*3 (7.7%) were found as the major FAs. Compared with the FA profile from krill oil, as shown in Table 1, both the *P. borealis* shrimp oil and the whole shrimp contain less SFAs and more unsaturated FAs. The content of ω-3 PUFAs in shrimp oil is close to the previous report from TAG rich fraction of krill oil (25%) [23], fish oil (27%) [36] and cod liver oil (24%) [27].

Potential health benefits of PUFAs and MUFAs derived from marine oils are reported to reduce body weight [37], promote lipid metabolism [38,39], control hyperlipidemia [40], prevent cardiovascular diseases [41,42], circulate lipids and reduce chronic inflammation [43,44,45]. The American Heart Association suggested that MUFAs have a favorable effect on cardiovascular disease risks [39,46] and may have benefit in preventing type-2 diabetes [47,48]. Since Northern shrimp oil contains significant amounts of both MUFAs and PUFAs, it may offer a unique health benefits that are worth further investigations.

### 3.2. Characterization and Structural Elucidation of TAGs in Shrimp Oil

A total of 118 TAG species has been identified by LC-HRMS and MS/MS in this work, 40 of which are with PUFAs on either *sn*-1,3 and/or *sn*-2 positions. Several abundant TAGs in the Northern shrimp oil include 16:0/16:1/16:1, 16:0/20:1/20:5, 16:0/18:1/20:5, 16:1/18:1/18:1, 16:1/16:1/20:1, 18:1/18:1/22:6, 16:0/16:0/20:5, 16:1/22:1/22:6, and 18:1/20:1/22:6. PUFAs (DHA and EPA) were on TAG backbones along with more MUFAs (16:1, 18:1, 20:1 and 22:1) than with SFAs (14:0 and 16:0).

Fatty acids can be distributed at three available positions (*sn*-1, *sn*-2 and *sn*-3) of a TAG molecule. Thus, analysis of TAGs in marine oils is quite challenging due to the very similar chemical and physical properties of their regioisomeric structures. Several studies have addressed the characterization of TAG in marine oils by using various techniques, including chemical/enzymatic hydrolysis, NMR and HRMS. However, enzymatic/chemical hydrolysis is complex, time-consuming and prone to contamination with hydrolysis byproducts [49,50,51]. Furthermore, due to its fatty acid selectivity, enzymatic analysis is not applicable to marine oils as specified in the official IUPAC method [52]. Typically, high resolution ^13^C-NMR and LC-HRMS methods present valuable techniques in the analysis of marine oils. For instance, NMR can provide information on the regiospecific distribution of FAs in TAG molecules [25,26,53]. LC-HRMS can be effectively employed for high-throughput lipids profiling and TAGs structural elucidation directly on complex oils [23,24,28].

Full elucidation of TAG structures requires not only the characterization of individual FA substituents but also the determination of the position of the acyl groups on the glycerol backbone. ESI-MS/MS has been previously applied to probe the preferential cleavage fragmentation mechanisms of TAG positional isomers [54,55,56]. Characterization of the acyl group disposition on the TAG molecules can be determined based on the intensity of DAG fragment ions produced from MS/MS spectra. Zeng *et al.* used pure TAG standards, 1-Arachidin-2-olein-3-palmitin-glycerol (AOP), 1-arachidin-2-palmitin-3-olein-glycerol (APO), 1-palmitin-2-arachidin-3-olein glycerol (PAO), 1-arachidin-2-linolein-3-olein-glycerol (ALO) and 1-palmitin-2-olein-3-linolein-glycerol (POL) to trace the relative intensities of product ion signals to the different positions of FAs on TAG molecules [27]. The TAG molecule containing three different acyl groups gave rise to three DAG fragments, and the least abundant DAG fragment ion corresponded to the loss of fatty acid from the middle position (*sn*-2). The results indicated that the neutral loss of *sn*-2 fatty acid was energetically less favorable than loss of the fatty acid from the *sn*-1 or *sn*-3 position. It could be that the loss of external FAs (*sn*-1 or *sn*-3), which leads to a six member ring, is more stable compared to the loss of FAs at *sn*-2 that leads to the formation of a five member ring [28]. However, Baiocchi *et al.* suggested that this interpretation is not useful for complex oils containing high amount of omega-3 long chain-PUFAs on TAGs [28]. They compared thermal- and collision- induced fragmentation of TAGs in tuna oil and algae oil. The relative intensity of the MS/MS signals corresponding to the loss of FAs induced by both modes resulted in different ion abundance profiles. The fragmentations induced by collisional dissociation were contradictory. Among three DAG fragment ions of the MS/MS spectrum, the most abundant DAG fragment ion corresponded to the loss of fatty acid from the *sn*-2 position. Characterization of FAs position on TAG backbones by LC-MS/MS has been applied to elucidate the structural isomers of terrestrial plant [57,58] and marine oils [23,27]. However, the co-eluting TAGs in the shrimp oil yielded multiple combinations of DAG fragment ions. Thus, those interpretative schemes may not be used alone when complex samples are involved.

The regioisomeric distribution of FAs on TAGs in this Northern shrimp oil was then determined by ^13^C-NMR. Only major FAs profiled in GC including SFA (16:0) and MUFAs (16:1, 18:1, 20:1 and 22:1), EPA (20:5) and DHA (22:6) are included (Table 3). The regioisomeric distribution (molar %) of major FAs obtained was consistent to those obtained by GC analysis. For example, the total SFAs and MUFAs attached on TAGs (*sn*-1,3 + *sn*-2) was calculated to be 75.9% by ^13^C-NMR, compared with 69.6% by GC. Interestingly, SFAs and MUFAs appeared to be slightly higher at *sn*-1,3 positions than the *sn*-2 position (56.8% *vs.* 19.2%). As for PUFAs, both EPA and DHA were found preferably at the *sn*-2 position than *sn*-1,3 positions, with DHA predominately on *sn*-2 position. PUFAs are mainly located on the *sn*-2 position of TAG in shrimp oil, which is in accordance with the finding for cod liver oil [27], tuna oil [28] and algae oil [28], as outlined in Table 3. However, Suárez *et al.* found EPA in fish oil located primarily at the *sn*-1,3 position, whereas most of DHA is at the *sn*-2 position [25].

These structural differences of TAG species may affect their stability and bioavailability. There are reports that the oxidative stability of TAGs with unsaturated fatty acids at *sn*-2 positions was higher than that at *sn*-1,3 positions [59,60,61]. Although it is still debatable, some studies have suggested that the absorption of FAs at *sn*-2 positions is more favored [28,62,63]. Thus, the Northern shrimp oil with PUFAs preferentially attached to the *sn*-2 positions of the TAG may present higher stability resulting in a favorable absorption of omega-3 FAs relative to other forms, which would be of benefit for use as a health supplement ingredient.

### 3.3. Characterization and Identification of Astaxanthin Species of Shrimp Oil

The astaxanthin molecule, possessing two hydroxyl functions located at C3/C3′ of the β-ionone moieties, may be present in its free form as well as in its mono- or di-esterified form depending on the sources. Based on the chemical and physical properties of astaxanthin, HPLC with diode-array detection has become a common analytical method for qualitative and quantitative determination of astaxanthin [64,65,66]. More recently, researchers have complemented the identification of astaxanthin and its esters using other specific detection methods, including HPLC coupled with mass spectrometry (LC-MS) [31,66,67], nuclear magnetic resonance spectroscopy (NMR) [33,68] and Raman spectroscopy [69,70].

The Northern shrimp oil obtained as byproducts from shrimp processing facilities has a bright red color attributed to astaxanthin. The concentration of astaxanthin in the shrimp oil was found to be more than 800 mg/kg [71], compared to 14 to 39 mg/kg in wet shrimp shells [3]. In contrast to microalga *Haematococcus pluvialis,* which principally contains singly esterified astaxanthin [31,68], di-esters formed the main components in shrimp oil. These results are in accordance with those presented by Breithaupt [31].

As for its fatty acid composition, Renstrøm and Liaaen-Jensen [72] reported that astaxanthin mono- and di-esters from shrimp (*P. borealis*) contained only even-numbered fatty acids: saturated (C12:0, C14:0, C16:0, C18:0, about 13% of total), mono-unsaturated (C16:1, C18:1, C20:1, C22:1, about 70%) and poly-unsaturated (C18:2, C20:5, C22:6) based on GC analysis after saponification. In their study, C18:1 and C22:1 formed the predominant components while some other PUFAs were also present in significant amounts. In another study, the composition of astaxanthin esters in methanol/ethyl acetate/light petroleum (1:1:1, *v*/*v*/*v*) extract from shrimp (*P. borealis*) was determined by negative ion liquid chromatography-atmospheric pressure chemical ionization mass spectrometry [31], with five mono-esters and eight di-esters identified. Lauric acid (C12:0) was found to be the prevalent fatty acid; however, PUFAs were not found as main components in that work. In our study, lauric acid (C12:0) and oleic acid (C18:1) have been found to be the most abundant fatty acids esterified with astaxanthin esters in shrimp oil. Moreover, astaxanthin species esterified with EPA and DHA present 30.5% of total astaxanthin compounds in shrimp oil. Additionally, seven new astaxanthin compounds mainly esterified with unsaturated FAs were identified in this study.

The components of astaxanthin esters vary from species to species in shrimps. Yang *et al.* identified 26 free astaxanthin and its fatty acids esters from raw Pacific white shrimp *Litopenaeus vannamei*, 32.95% of which were recognized as free astaxanthin, 58.65% as mono-esters and only 8.4% as di-esters [32]. Their work also revealed that astaxanthin diesters had a better thermostability than monoesters. There is a hypothesis that the stability of astaxanthin compounds during storage and thermal processing remains: diesters > monoesters > free astaxanthin [73]. The possible reasons are (1) the longer chain of diesters molecules may require higher energy to break up; and (2) the diesters may have better integration with biological matrices due to their lower polarity and greater lipophilic property that may protect them from degradation [73,74]. Thus, astaxanthin in the Northern shrimp oil may have higher stability than that in other marine oils and warrants further study.

## 4. Materials and Methods

### 4.1. Materials

Astaxanthin was purchased from ChromaDex Inc. (Irvine, CA, USA). (rac./meso)-Astaxanthin dipalmitate ((3*RS*,3′*RS*)-3,3′-dihexadecanoyloxy-β,β-carotene-4,4′-dione) was purchased from CaroteNature GmbH (Ostermundigen, Bern, Switzerland).

Individual lipid standards for HPLC including fatty acids (Myristic acid, *cis*-Palmitoleic acid, *cis*-Vaccenic acid, *trans*-Vaccenic acid, Oleic acid, Elaidic acid, Pentadecanoic acid, Palmitic acid, Heptadecanoic acid, *cis*-10-Heptadecenoic acid, Stearic acid, 9-*cis*,12-*cis*-Linoleic acid, *cis*,*cis*,*cis*-9,12,15-Octadecatrienoic acid, *cis*,*cis*,*cis*-6,9,12-Octadecatrienoic acid, *cis*-11-Eicosenoic acid, Arachidonic acid, *cis*-5,8,11,14,17-Eicosapentaenoic acid, *cis*-4,7,10,13,16,19-Docosahexaenoic acid, and Tricosanoic acid), monoglycerides (dl-α-Palmitin, 1-Monopalmitoleoyl-*rac*-glycerol, and 1-Oleoyl-*rac*-glycerol), Phospholipids mixer (l-α-Lysophosphatidylcholine, l-α-Phosphatidylcholine, l-α-Phosphatidylethanolamine, and l-α-Phosphatidylinositol sodium salt) and triglycerides (Olive oil) were purchased from Sigma-Aldrich Inc. (St Louis, MO, USA).

All solvents used for mass spectrometry were of HPLC-grade purchased from Caledon Laboratories Ltd. (Georgetown, Ontario, Canada), and other chemicals were of analytical grade.

Shrimp oil was obtained with an extraction process that is patent pending [71]. Briefly, Northern shrimp (*P. borealis*) processing water was recovered and subjected to a dissolved air flotation system after adding a flocculating agent. The suspended and dissolved solids formed aggregates that were recovered from the surface, and was then directed into a horizontal centrifuge in order to separate the solid phase and the liquid phase consisting of water and oil. The liquid phase was pumped into the 3-phase vertical centrifuge in order to separate the shrimp oil, the water, and the solids.

A clean up procedure for shrimp oil was adapted from the literature [75]. The Supelclean™ LC-Si SPE cartridge (Sigma-Aldrich Corp., St Louis, MO, USA) was washed with 5 mL hexane. Ten milligrams of shrimp oil dissolved in 5 mL hexane were loaded into a pre-treated SPE cartridge. The column was eluted with 2 × 5 mL hexane containing 6% diethyl ether to obtain a triacylglycerol rich fraction and then with 2 × 5 mL acetone containing 200 mg/L BHT to obtain the pigments. The two acetone fractions were pooled and were evaporated under a flow of nitrogen and kept at −20 °C until they were analyzed.

### 4.2. High Performance Liquid Chromatography (HPLC)

HPLC was carried out on an 1100 series instrument (Agilent Technologies, Santa Clara, CA, USA) with a Thermo Scientific™ Dionex™ Corona™ Charged Aerosol Detector (CAD) (Dionex Company, Chelmsford, MA, USA) and a diode array detector (DAD, 476 nm) (Agilent Technologies, Santa Clara, CA, USA). The separations were performed on a HALO C8 column (2.1 mm × 100 mm, 2.7 μm, Advanced Materials Technology Inc., Wilmington, DE, USA) with a 0.45 mL/min flow rate at 40 °C. A 5 × 2.1 mm C8 cartridge (2.7 μm, Advanced Materials Technology Inc.) was used as the pre-column. The separation of the shrimp oil (*v*/*v* 1:100 with isopropanol) was achieved using a mobile phase composition (gradient 1) of (A) Methanol/water/acetic acid (750:250:4, *v*/*v*/*v*), and (B) Acetonitrile/methanol/tetrahydrofuran/acetic acid (500:375:125:4, *v*/*v*/*v*/*v*). The elution started with a liner gradient from 0% B to 70% B in 40 min at 0.8 mL/min, then went up to 90% B in 10 min, and finally reached to 100% B in another 10 min, holding for 20 min, then back to 0% B in 1 min and held for 10 min. Lipid standards were made to 1 mg/mL, and applied on the column under same conditions. The injection volume was 10 μL.

Gradient 2 for astaxanthin elution was using (A) 50 mM ammonium bicarbonate, (B) methanol, (C) isopropanol, and (D) acetonitrile. The elution was proceeded at 18% A, 80% B and 2% D for 27 min, followed by 90% B and 10% D for 8 min, then changed to 28% B, 70% C and 2% D within 2 min and held for 5 min.

### 4.3. FAME Preparation

FAs in shrimp oil were converted into FAMEs formed according to the protocol provided by Sigma-Aldrich Inc. with modification [76]. Shrimp oil was hydrolyzed with 1.5 N NaOH methanol solution under N_2_ at 100 °C for 5 min, then methylated with 14% of BF_3_ methanol solution at 100 °C for 30 min. Distilled water was added to stop the reaction. Methylated fatty acids were extracted with hexane and injected on to the column.

### 4.4. Gas Chromatography (GC)

GC analysis was carried out on an Agilent Technologies 7890A GC spectrometer (Agilent Technologies, Santa Clara, CA, USA), using an Omegawax 250 fused silica capillary column (30 m × 0.25 mm × 0.25 μm film thickness, Sigma-Aldrich Inc.). The carrier gas was ultra-pure helium at a flow rate of 3 mL/min. The column was set at 185 °C, held for 4 min, then ramped to 230 °C at a rate of 3 °C/min and held for 7 min. The injector temperature was 260 °C. The detector was held at 260 °C. Supelco^®^ 37 component fatty acid methylated esters mix and PUFA-3 (Supelco, Bellefonte, PA, USA) were used as fatty acid methyl ester standards. The FAME profiles of samples were identified by comparison of their retention times with that of FAME standards. The peak areas were used for concentration calculation.

### 4.5. LC-MS Instrumentation

Screening for astaxanthin esters and TAGs was carried out on an Exactive™ Orbitrap instrument (Thermo Fisher Scientific, Waltham, MA, USA), a high resolution mass spectrometer, based on a method by Békri *et al.* [77]. A Thermo Accela 1250 (Thermo Fisher Scientific, Waltham, MA, USA) quaternary LC system was coupled to the Exactive™ (Thermo Fisher Scientific, Waltham, MA, USA) equipped with a heated electrospray ionization probe HESI-II (Thermo Fisher Scientific, Waltham, MA, USA). LC separation was performed on an HALO C8 column (2.1 × 100 mm, 2.7 µm, Advanced Materials Technology Inc.) at 30 °C using the following mobile phases: (A) 50 mM ammonium acetate; (B) methanol; (C) isopropanol and (D) acetonitrile. Analytes were eluted with a linear gradient from 18:80:0:2 (A/B/C/D) to 0:90:0:10 (A/B/C/D) in 27 min, held at this composition for 8 min, and then to 0:28:70:2 (A/B/C/D) in 2 min, held at 5 min, and returning to initial composition in 1 min. The flow rate was 500 µL/min. Ion source conditions consisted of a spray voltage of 3 kV, sheath flow of 55, auxiliary gas flow of 15, capillary temperature of 380 °C and heater temperature of 400 °C. Positive polarity scans were acquired at alternating MS scan of 0.5 s (2 Hz) and higher energy collisional dissociation scan of 0.1s (10 Hz) operating at 65 eV. Acquistion was carried out using Xcalibur 2.1 (Thermo Fisher Scientific, Waltham, MA, USA). To facilitate the astaxanthin identification process, a list of theoretical astaxanthin mono-esters and di-esters masses with carbon numbers of free fatty acid chains from 8 to 30 was created. Entities were extracted using ToxID 2.1.2 (Thermo Fisher Scientific, Waltham, MA, USA) based on the criteria of a 5 ppm mass window centered on the target analytes. The mass spectrum of each analyte peak was manually inspected to rule out false positive. For TAG identification, a data file of glycerols esterified with FAs downloaded from LipidMAPS database was modified with a variety of custom fatty acids. This data file was used for screening for the presence of TAGs in shrimp oil in ToxID software automatically based on the exact masses (<5 ppm from theoretical mass).

To confirm the preliminary results obtained on Exactive™ Orbitrap instrument (Thermo Fisher Scientific, Waltham, MA, USA), MS/MS spectra of individual astaxanthin analytes were acquired on an Acquity ultra performance liquid chromatography (UPLC) system coupled to a Quadrupole time-of-flight (Q-TOF) Premier mass spectrometer (Waters Corp., Milford, MA, USA) equipped with an electrospray ionization LockSpray™ modular source (Waters, Milford, MA, USA). A lock mass solution of 33 pg/µL leucine enkephalin was delivered continuously to correct for drifts in mass measurements. Separation was performed on the same analytical column at 40 °C using a modified method from McNichol *et al.* [78]. The mobile phases composed of (A) 50 mM ammonium acetate/methanol 20/80 (*v*/*v*) and (B) isopropanol/methanol 70/30 (*v*/*v*). Analytes were eluted with a linear gradient from 100% A to 100% B in 50 min, held at 100% B for 5 min, and returning to 100% A in 1 min with a flow-rate of 450 µL/min. Capillary voltage was at 3.2 kV, cone voltage of 32 V, collision energy of 25 eV, source and desolvation temperature at 120 °C and 400 °C, respectively. For TAG identification, data dependent acquisition was acquired in order to maximize the number of tandem MS spectra. The survey scan switched to MSMS when the intensities arise above a threshold of 50 counts/s. Three MSMS channels were selected, each with scan time set at 0.2 s. The MSMS automatically switched back to survey after 2 s, or when the intensity felt below 100 counts/s. Collision energy was set at 30 eV. Acquisition was carried out using MassLynx 4.1 (Waters, Milford, MA, USA). The relative abundance of each analyte was calculated based on its peak area, with the assumption they have the same ionization efficiency.

### 4.6. ^13^C-NMR Spectroscopy

^13^C NMR spectra were acquired of 200 mg of shrimp oils in 99.8% CDCl_3_ at 20°C using a Bruker AV-III 700 MHz spectrometer equipped with a 5 mm TCI cryoprobe using Topspin V2.1 (Bruker Biospin, Milton, ON, Canada) software. The data were acquired at a ^13^C frequency of 176.05 MHz into 128K complex time domain points using a 90 degree pulse and a recycle time of 16.6 s between pulses. The spectra were acquired with ^1^H decoupling using the WALTZ16 pulse sequence and an inverse gated decoupling scheme to suppress NOE effects [25]. The spectra were signal-averaged over 256 scans with 4 dummy scans to achieve equilibrium conditions. Prior to Fourier transform the free induction decays (FID) were zero filled to 128K real points and filtered using an exponential window function with a line broadening parameter of 0.3 Hz.

The regioisomeric distributions of major fatty acids on TAGs molecules in shrimp oil were calculated by determining the resonance areas using the standard deconvolution algorithm in Topspin 2.1 assuming a pure Lorentzian lineshape. The regioisomeric distributions (molar %) were expressed as the percentage of *sn*-1,3 (or *sn*-2) chains over the total chains (*sn*-1,3 + *sn*-2).

## 5. Conclusions

The health benefits of marine oils have generated interest for an in-depth understanding of the structural features of its lipid components. An analytical approach using HPLC, HRMS and MS/MS is presented for characterization of TAGs and astaxanthin fatty acid esters from Northern shrimp oil, which provides reliable identification of individual fatty acid esters without time-consuming isolation or derivatization. Meanwhile, the lipid classes and FA profiles of the shrimp oil and their specific positions on intact TAG molecules have been successfully confirmed for the first time by HPLC, GC, LC-HRMS and ^13^C-NMR analysis. Based on the present study, the shrimp oil contained 49.9% MUFAs, 19.7% SFAs and 29.2% PUFAs. Moreover, 16:1*n*7, 16:0, 18:1*n*9, 20:5*n*3 (EPA), 22:6*n*3 (DHA) and 22:1*n*9 were found to be the most abundant fatty acids. LC-HRMS and MS/MS strategies were developed to directly characterize the acyl groups on the TAG and astaxanthin esters of shrimp oil. A total of 118 TAGs mainly esterified with unsaturated fatty acids such as EPA and DHA were elucidated as the major composition of the shrimp oil. The characterization of TAGs at a molecular level also revealed that PUFAs were on TAGs with more MUFAs than with SFAs. It also showed that PUFAs were preferably distributed at the *sn*-2 position. In addition, 38 astaxanthin compounds including three free astaxanthin, nine mono-esters and 26 di-esters were identified in shrimp oil. Among them, seven astaxanthin compounds that have not been identified from shrimp before were reported in this study. In conclusion, detailed characterization of shrimp oil has provided not only the total composition of FAs, but structural information of intact TAGs and astaxanthin esters in shrimp oil. Characterization of the key components of Northern shrimp oil presented here provides an important basis for a more complete understanding of the healthful benefits of this oil as a viable health supplement or food ingredient.

## Supplementary Files

Supplementary File 1
