# Supplementary material for: Characterization of Shrimp Oil from Pandalus borealis by High Performance Liquid Chromatography and High Resolution Mass Spectrometry
Source: Mar Drugs. 2015 Jun 18;13(6):3849–76. doi: 10.3390/md13063849 (PMC4483660; doi:10.3390/md13063849)
Supplement: Supplementary File 1 [file marinedrugs-13-03849-s001.pdf]

Supplementary Information

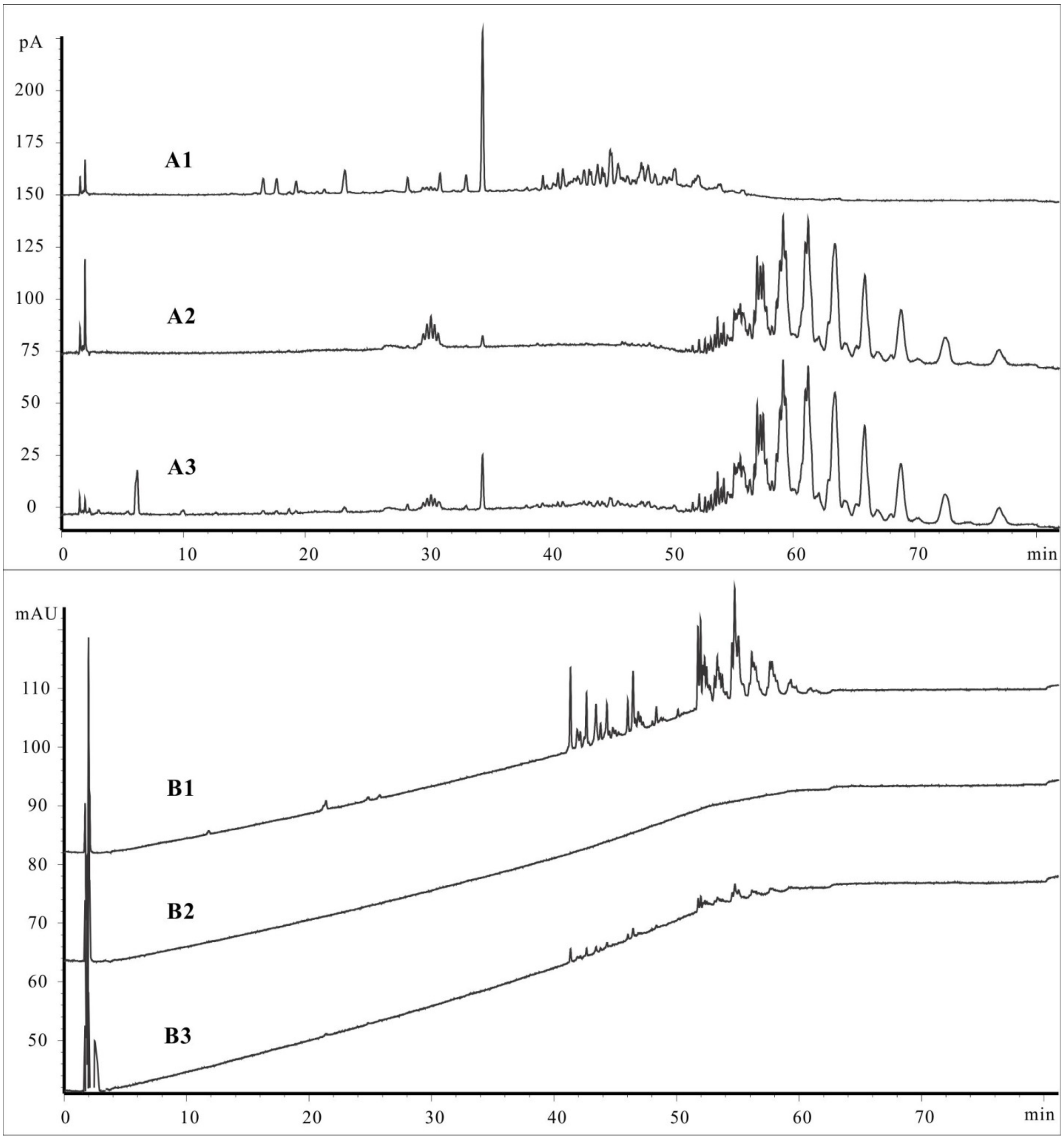

Figure S1. Chromatograms of two fractions of shrimp oil after SPE cleanup, monitored by CAD detector (**A**) and DAD 476 nm detector (**B**). HPLC was performed on an HALO C8 column eluted with gradient 1. **A1** and **B1** present astaxanthin rich fraction on CAD and DAD chromatograms, respectively. **A2** and **B2** are those of triacylglycerol rich fraction, whereas **A3** and **B3** are those of shrimp oil. Compared with three CAD chromatograms as shown in **A1**, **A2** and **A3**, triacylglycerol were totally separated from the astaxanthin fraction. It is evident that the DAD chromatogram of triacylglycerol fraction (**B2**) showed no pigment. Taken together, SPE cleanup experiment successfully removed all the triacylglycerol apart from shrimp oil and left astaxanthin rich fraction for MS analysis.

© 2015 by the authors; licensee MDPI, Basel, Switzerland. This article is an open access article distributed under the terms and conditions of the Creative Commons Attribution license (http://creativecommons.org/licenses/by/4.0/).
